# The Liver as a Biomarker Organ in Heart Failure: Molecular Mechanisms, Hepatic Scores and Systemic Risk Stratification

**DOI:** 10.3390/ijms27177545

**Published:** 2026-08-23

**Authors:** Wioletta Szczurek-Wasilewicz, Antoni Borowiec, Iga Waluszewska, Bożena Szyguła-Jurkiewicz

**Affiliations:** 1 Department of Pharmacology, Faculty of Medicine, University of Opole, 45-052 Opole, Poland; 2 Students’ Scientific Society, Department of Pharmacology, Faculty of Medicine, University of Opole, 45-052 Opole, Poland; antoni.borowiec2@gmail.com (A.B.); igawaluszewska@gmail.com (I.W.); 3 Faculty of Medicine, University of Opole, 45-052 Opole, Poland; bjurkiewicz@sum.edu.pl; 4 3rd Department of Cardiology, Faculty of Medical Sciences in Zabrze, Medical University of Silesia, 40-055 Katowice, Poland

**Keywords:** heart failure, liver biomarkers, cardiohepatic syndrome, congestive hepatopathy, hypoxic hepatitis, hepatic scores, left ventricular assist device, heart transplantation

## Abstract

Heart failure (HF) is a systemic syndrome in which prognosis depends on cardiac dysfunction, congestion, cardiorenal and cardiohepatic interactions, inflammation, and metabolic dysregulation. The liver is exposed to elevated systemic venous pressure and reduced forward flow, and contributes to albumin and coagulation factor synthesis, bile acid metabolism, iron homeostasis, and the acute-phase response. Cardiohepatic injury involves hemodynamic stress, sinusoidal endothelial dysfunction, oxidative stress, inflammatory signaling, fibrogenesis, and altered metabolic regulation. Congestive hepatopathy is associated with right-sided HF, tricuspid regurgitation (TR), pulmonary hypertension, and elevated central venous pressure, whereas hypoxic hepatitis develops during low-output states, shock, or acute circulatory deterioration. These mechanisms may coexist, producing congestive/cholestatic, hypoperfusive/ischemic and mixed/systemic reserve profiles. Composite liver-related scores, including Model for End-Stage Liver Disease (MELD), MELD excluding International Normalized Ratio (MELD-XI), MELD with sodium (MELD-Na), MELD-Albumin and albumin–bilirubin (ALBI) score, may reflect congestion, hepatorenal dysfunction, nutritional status and reduced systemic reserve. This review summarizes hemodynamic and molecular mechanisms of cardiohepatic injury, liver-related biomarkers and composite scores, with emphases on advanced HF, left ventricular assist device (LVAD) therapy and heart transplantation. Liver-related abnormalities remain underrecognized in HF. Their serial interpretation may support risk stratification, but composite scores should complement rather than replace comprehensive clinical assessment.

## 1. Introduction

Heart failure (HF) is a systemic syndrome in which the clinical course depends not only on cardiac dysfunction, but also on congestion, cardiorenal and cardiohepatic interactions, inflammatory activation, and metabolic dysregulation [1,2,3]. This is particularly relevant in advanced HF, where prognosis is rarely explained by left ventricular dysfunction alone. The kidneys, liver, lungs, skeletal muscles, intestine, and vasculature contribute to disease progression and influence symptoms, treatment tolerance, and long-term outcomes [1,2].

The liver has a distinct role in this systemic process because it is exposed to both increased systemic venous pressure and reduced forward flow. It also regulates albumin and coagulation factor synthesis, bile acid metabolism, iron homeostasis, and the acute-phase response. Cardiohepatic interactions in HF are therefore not limited to abnormal liver tests, but represent broader relationships between hemodynamic stress, hepatic metabolism and systemic risk [2,4].

Increased right atrial pressure is transmitted to the inferior vena cava, hepatic veins and hepatic sinusoids, leading to hepatic congestion and, in many patients, cholestatic liver injury [2,4,5]. Reduced cardiac output (CO) may impair hepatic perfusion and oxygen delivery, especially during acute decompensation, cardiogenic shock or severe circulatory deterioration [1,4,5]. In clinical HF, these mechanisms rarely occur in isolation, as venous congestion may impair effective hepatic microcirculatory perfusion and increase susceptibility to hypoxic hepatocellular injury even without severe systemic hypotension [4,5].

Abnormal liver tests are common in HF and may provide prognostic information [6,7,8]. Increased bilirubin and cholestatic enzymes usually reflect congestion and impaired bile transport, whereas marked aminotransferase elevation suggests acute hepatocellular injury related to hypoperfusion [5,7,8]. Low albumin may indicate impaired hepatic synthesis, but in HF it is also influenced by inflammation, malnutrition, hemodilution and protein loss [6,8]. Therefore, liver abnormalities should not be interpreted only as routine biochemical findings or as isolated markers of hepatic disease. Even mild abnormalities in bilirubin, albumin or cholestatic enzymes may identify patients with more advanced congestion, renal dysfunction, impaired nutritional status and higher risk of adverse outcomes [6,7,8]. This is especially important in advanced HF, left ventricular assist device (LVAD) therapy and heart transplantation, where hepatic and hepatorenal reserve may influence perioperative risk, therapeutic strategy and prognosis [9].

Composite liver-related scores have gained increasing interest in HF since they provide an integrated estimate of cardiohepatic, cardiorenal and systemic vulnerability based on routinely available laboratory parameters. Model for End-Stage Liver Disease (MELD) and Model for End-Stage Liver Disease Excluding International Normalized Ratio (MELD-XI) combine bilirubin with renal indices, while MELD additionally includes International Normalized Ratio (INR) and MELD-XI avoids confounding by anticoagulation [9]. The albumin–bilirubin score (ALBI) adds an INR-independent assessment based on bilirubin and albumin, capturing elements of congestion, hepatic reserve, nutritional status and systemic inflammation [10]. Thus, in HF, the prognostic relevance of these scores should not be interpreted as a marker of liver dysfunction alone, but rather as a composite signal of hepatorenal impairment, venous congestion and reduced physiological reserve.

Although cardiohepatic interactions have been recognized for decades, the traditional literature has largely viewed hepatic involvement as a passive downstream consequence of venous circulatory failure. However, emerging evidence supports a broader interpretation of the liver as an integrator of hemodynamic, renal, inflammatory and metabolic abnormalities.

Existing reviews have largely focused on individual mechanisms, often without fully integrating emerging molecular insights with their clinical implications. In contrast, this review provides a critical and contemporary overview linking novel molecular pathways, including inflammation-driven hepcidin dysregulation, disruption of the gut–liver axis, hepatokine signaling, and extracellular vesicle transport, with clinical interpretation and risk assessment. Furthermore, this narrative review summarizes the diagnostic and prognostic value of classic biomarkers and INR-independent liver scores (MELD-XI, ALBI) across the spectrum of heart failure, from acute and chronic decompensation to advanced management strategies, including LVAD therapy and heart transplantation.

## 2. Search Strategy and Methods

This review focuses on cardiohepatic interactions, liver-derived biomarkers and composite hepatic scoring systems in HF. A comprehensive literature search was conducted across the PubMed, Web of Science, and Scopus databases to identify relevant original articles, clinical studies, narrative and systematic reviews, and professional society guidelines. The search was last updated in August 2026 and covered publications from January 1999 to that date. Only peer-reviewed publications in English were considered. Clinical evidence—particularly human cohort studies, clinical registries, and international management guidelines (e.g., guidelines from the International Society for Heart and Lung Transplantation (ISHLT) 2024) evaluating patient outcomes in advanced HF, LVAD support, and heart transplantation—was prioritized over experimental findings. Mechanistic and animal studies were selectively included to provide essential pathophysiological context regarding sinusoidal stress, oxidative injury, and inflammatory pathways. Articles were screened based on title, abstract, and full-text relevance to cardiohepatic pathophysiology, prognostic scoring, or clinical management in HF. Studies focusing purely on primary non-cardiac liver pathology or conference abstracts lacking complete methodology were excluded.

## 3. Hemodynamic Basis and Clinical Interpretation of Liver Biomarkers in Heart Failure

Abnormal liver tests in HF should be interpreted within the hemodynamic and systemic profile of the disease. In advanced HF, hepatic abnormalities rarely result from a single mechanism. Venous congestion and reduced forward flow contribute to liver injury [2,3,4,5]. Renal dysfunction, inflammatory activation and impaired nutritional status often coexist, contributing to the overall biochemical profile [1,6,7,8,11]. For this reason, the clinical value of liver-derived biomarkers lies not in their specificity for liver disease, but in their ability to reflect congestion, perfusion abnormalities and systemic severity.

The liver is particularly vulnerable to elevated systemic venous pressure. Increased right atrial pressure is transmitted to the inferior vena cava, hepatic veins and hepatic sinusoids, causing sinusoidal congestion and impaired hepatic venous outflow [2,4,5,12]. Because hepatic sinusoids are low-pressure vascular channels, even moderate venous pressure elevation may disturb microcirculatory flow and reduce oxygen diffusion to hepatocytes. The centrilobular region, especially zone 3, is most susceptible to injury because of its lower physiological oxygen tension. Chronic congestion may therefore lead to pathological changes, such as sinusoidal dilatation, centrilobular hepatocyte injury, hepatocyte atrophy and perisinusoidal fibrosis [4,12].

Congestive hepatopathy is not only a passive consequence of elevated venous pressure. Persistent congestion may promote sinusoidal endothelial dysfunction, Kupffer cell activation and hepatic stellate cell stimulation. These processes contribute to local inflammation, extracellular matrix deposition and progressive hepatic remodeling [12]. This may explain why structural liver injury can develop in long-standing right-sided HF even when routine liver tests remain only mildly abnormal.

The biochemical profile of venous congestion is usually cholestatic. Increased bilirubin, gamma-glutamyl transferase (GGT) and alkaline phosphatase (ALP) may reflect impaired sinusoidal drainage, disturbed bile transport and reduced hepatic clearance [4,5,12,13]. Aminotransferases are often normal or only mildly elevated. In clinical cohorts, cholestatic abnormalities and bilirubin elevation have been associated with worse outcomes in HF [6,7,8]. Thus, in patients with right ventricular (RV) dysfunction, severe tricuspid regurgitation (TR), hepatomegaly, ascites, or peripheral edema, even moderate hyperbilirubinemia may indicate clinically relevant systemic congestion and higher risk [2,5,8,12]. Reduced CO represents the second major mechanism of hepatic injury in HF [2,4,14]. Although the liver has a dual blood supply, adequate systemic perfusion remains essential for hepatocyte function. Severe or prolonged reduction in CO may cause acute hepatocellular injury, which is most clearly expressed as hypoxic hepatitis [14]. This condition is characterized by a rapid and marked increase in aminotransferases, often accompanied by high lactate dehydrogenase activity, and may occur in cardiogenic shock, severe acute decompensation, cardiac arrest or other states of critical circulatory failure [1,14].

In HF, venous congestion and hypoperfusion frequently interact. Elevated venous pressure impairs hepatic outflow, whereas reduced forward flow limits oxygen delivery. As a result, effective hepatic perfusion may decrease even without profound systemic hypotension [2,5,14]. This interaction is particularly important in advanced HF and RV dysfunction, where hepatic congestion increases susceptibility to ischemic injury.

RV dysfunction is central to cardiohepatic injury, determining the degree of systemic venous pressure transmitted to the hepatic circulation. This mechanism is particularly relevant in pulmonary hypertension, severe TR, restrictive cardiomyopathy and advanced biventricular HF [2,4,5,12]. It is also important in patients referred for LVAD implantation. Although LVAD therapy unloads the left ventricle and improves systemic forward flow, persistent or worsening RV failure may maintain hepatic congestion despite improved left-sided hemodynamics [9,15]. These mechanisms explain why single liver biomarkers should not be interpreted in isolation. Increased bilirubin may reflect venous congestion, impaired bile transport or reduced hepatic clearance [4,5,12,13]. Marked aminotransferase elevation usually suggests acute circulatory deterioration [14]. Low albumin may indicate impaired hepatic synthetic function, but in HF it more often reflects the combined effects of inflammatory activation, impaired nutritional status, fluid overload with hemodilution, and renal or gastrointestinal (GI) protein loss [11]. Thus, liver-derived biomarkers should be interpreted together with RV function, TR, pulmonary pressures, renal function, sodium concentration, congestion status and current treatment, particularly diuretic and anticoagulant therapy [2,5,12,16].

This hemodynamic complexity provides the rationale for using composite liver-related scores in HF. MELD, MELD-XI, Model for End-Stage Liver Disease with Sodium (MELD-Na), MELD-Albumin and ALBI do not identify a single mechanism of liver injury [9,10,16,17]. MELD-based scores integrate bilirubin, creatinine and, in selected formulas, INR, sodium, or albumin [9,16,17]. ALBI summarizes the albumin–bilirubin component and provides an INR-independent assessment of liver-related systemic risk [10,18]. Their clinical value is most evident in advanced HF, LVAD therapy and heart transplantation, where hepatic and renal abnormalities may influence perioperative risk, therapeutic strategy and prognosis [9,15,16,17].

The main hemodynamic and systemic mechanisms of cardiohepatic injury and their laboratory correlates are summarized in Table 1.

## 4. Molecular Mechanisms Linking Liver Injury and Systemic Progression in Heart Failure

The strength of evidence differs across the mechanisms discussed below. Clinical studies support the relationship between hemodynamic abnormalities and liver injury in HF [2,4,5,12,14]. Human HF studies have also documented systemic inflammatory activation, iron deficiency and intestinal dysfunction [19,20,21,22,23,24]. Direct evidence in human HF studies for specific hepatic molecular and cellular pathways remains limited. Evidence for sinusoidal endothelial and stellate-cell activation, interleukin−6 (IL-6)–hepcidin signaling and bile-acid pathways is derived mainly from experimental or non-HF studies [25,26,27,28,29,30], whereas hepatokines, extracellular vesicles and microRNAs have been discussed in cardiovascular (CV) and HF research but remain insufficiently validated clinically [31,32,33,34,35,36].

In HF, persistent venous congestion, intermittent hypoperfusion and tissue hypoxia may initiate sinusoidal and hepatocellular injury. Subsequent endothelial dysfunction, oxidative and inflammatory activation, altered iron and metabolic regulation and fibrogenesis may contribute to progressive systemic involvement [2,4,5,12,19,20,21,22,23,24,25,26,27,28,29,30,31,32,33,34,35,36,37,38]. Experimental and histopathological studies suggest that the hepatic sinusoid is an important site at which hemodynamic stress may be converted into cellular injury. These studies indicate that increased venous pressure can alter sinusoidal flow and impair oxygen diffusion, whereas hypoxia and oxidative stress may reduce nitric oxide (NO) bioavailability and promote endothelial activation [12,27,38]. These changes are most pronounced in zone 3 of the hepatic lobule, where physiological oxygen tension is lowest. Therefore, chronic congestion may produce microvascular injury and early remodeling even when routine liver tests are only mildly abnormal [4,5,12]. Oxidative stress further links cardiac dysfunction with hepatic injury. Oxidative stress is a well-recognized component of HF pathophysiology [37], whereas its effects on mitochondrial function, NO bioavailability and inflammatory signaling are supported mainly by experimental evidence [37,38]. In the liver, reduced oxygen delivery and impaired sinusoidal outflow increase hepatocyte susceptibility to ischemic injury. Acute severe hypoperfusion may lead to hypoxic hepatitis, whereas chronic oxidative stress may promote persistent low-grade hepatocyte injury and fibrogenesis [12,14,28,37]. In studies involving patients with HF, increases in circulating IL-6 and other inflammatory mediators have been associated with more advanced disease and adverse outcomes [19,20]. Experimental studies and research efforts in non-HF liver disease suggest that hypoxia, congestion and gut-derived signals may alter acute-phase protein synthesis and activate Kupffer cells and sinusoidal endothelial cells [12,27,28]. The extent to which this local hepatic response contributes to systemic inflammation in patients with HF is unclear.

**Figure 1:**
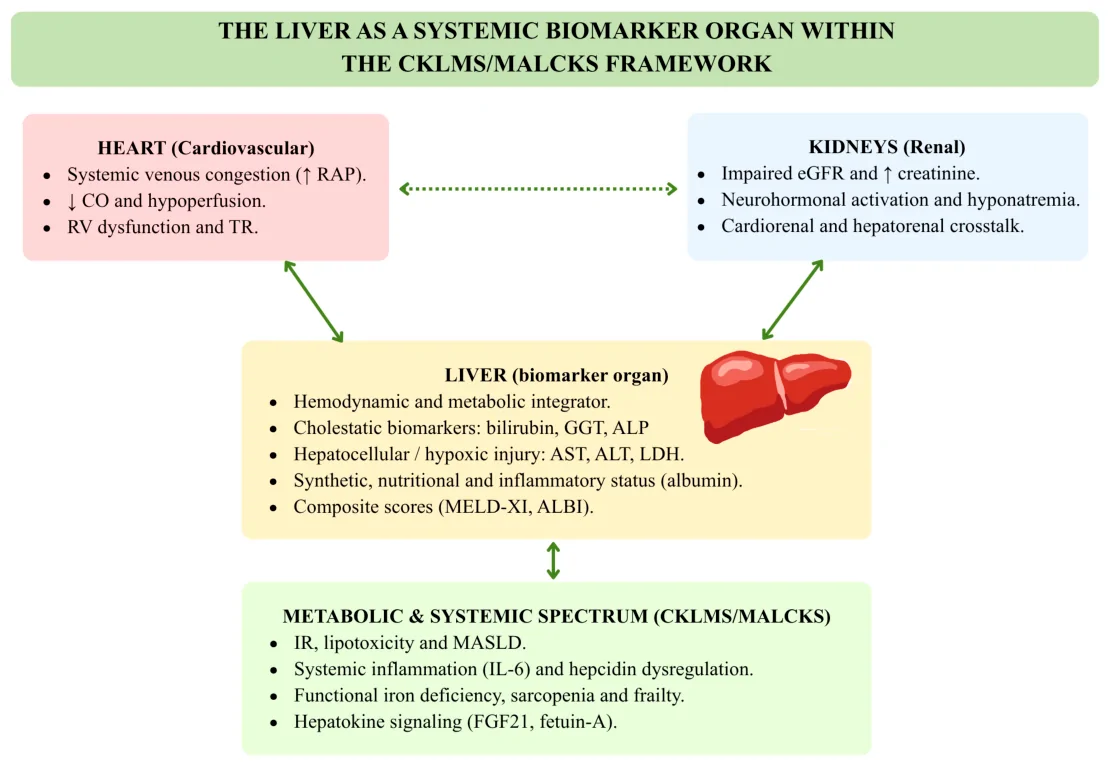
The liver as a systemic biomarker organ within the CKLMS/MALCKS framework. Double-headed arrows indicate bidirectional patophysiological interactions and clinical crosstalk between organ systems (cardiorenal, cardiohepatic, and hepatorenal axes), and the broader metabolic spectrum. Abbreviations: ALBI—Albumin-bilirubin Score; ALT—Alanine aminotransferase; ALP—Alkaline phosphatase; AST—Aspartate aminotransferase; CKLMS—Cardiovascular–Kidney–Liver–Metabolic Syndrome; CO—Cardiac output; eGFR—Estimated glomerular filtration rate; FGF21—Fibroblast growth factor 21; GGT—Gamma-glutamyl transferase; IL-6—Interleukin-6; IR—Insulin resistance; LDH—Lactate dehydrogenase; MALCKS—Metabolic-Associated Liver–Cardiovascular–Kidney Syndrome; MASLD—Metabolic dysfunction-associated steatotic Liver Disease; MELD-XI–Model for End-stage Liver Disease Excluding INR; RAP—Right atrial pressure; RV—Right ventricle; TR—Tricuspid regurgitation.

The IL-6–hepcidin–ferroportin pathway provides a possible link between inflammation and iron deficiency in HF. Hepcidin is produced mainly by hepatocytes and limits ferroportin-mediated iron export from enterocytes and macrophages [25]. Cellular studies have shown that IL-6 signaling increases hepcidin expression [26]. Although functional iron deficiency is common in HF and is associated with impaired exercise capacity and worse outcomes [21], the contribution of this hepatic pathway in patients with HF remains uncertain. Animal and cellular studies suggest that increased sinusoidal pressure, endothelial dysfunction, oxidative stress and local inflammation may activate hepatic stellate cells and promote extracellular matrix deposition [12,27,28]. Direct confirmation of this pathway in patients with HF remains limited. This process is clinically important, as structural liver remodeling may progress despite only modest changes in bilirubin, aminotransferases or cholestatic enzymes [4,5,12,28]. In advanced HF, this complicates the distinction between reversible congestion and established hepatic injury. The gut–liver axis may further amplify inflammation in advanced HF. Studies in patients with chronic HF have shown that venous congestion and reduced intestinal perfusion are associated with bowel wall edema, impaired barrier function and increased exposure to endotoxins [22,23,24]. Experimental evidence suggests that these signals may reach the liver through the portal circulation and activate Kupffer cells and sinusoidal endothelial cells [12,27,28]. This provides a plausible link between right-sided congestion and hepatic inflammation, and oxidative stress and systemic immune activation [12,22,23,24], although direct confirmation in patients with HF remains limited. Evidence for the role of bile acid signaling and hepatokines in HF comes mainly from preclinical studies and research conducted outside HF. Bile acids act through receptors such as farnesoid X receptor (FXR) and G-protein-coupled bile acid receptor 1 (TGR5) and may influence lipid, glucose and inflammatory pathways [29,30]. Hepatokines, including fibroblast growth factor 21 (FGF21), fetuin-A and selenoprotein P (SeP), are involved in insulin resistance, oxidative stress, vascular dysfunction and cardiometabolic disease [31,32,33]. These pathways may be relevant in HF patients with obesity, diabetes, chronic kidney disease (CKD), or metabolic dysfunction-associated steatotic liver disease (MASLD), but their clinical interpretation remains limited by low specificity [29,30,31,32,33]. Importantly, framing the liver as a biomarker organ directly aligns with emerging multi-organ paradigms, most notably Cardiovascular–Kidney–Liver–Metabolic Syndrome (CKLMS) and Metabolic-Associated Liver–Cardiovascular–Kidney Syndrome (MALCKS). These evolving frameworks reflect the profound pathophysiological crosstalk between cardiometabolic risk factors, CKD, and hepatic dysfunction—the last frequently manifesting as MASLD [39,40]. The position of the liver as a biomarker organ within the CKLMS and MALCKS frameworks is illustrated in Figure 1. Animal and cellular studies suggest that extracellular vesicles and circulating microRNAs may participate in organ communication by carrying proteins, lipids and non-coding RNAs involved in inflammation, fibrosis and metabolic regulation [34,35]. Liver-enriched microRNAs, especially miR-122, may reflect hepatocellular injury, whereas other circulating microRNAs have been linked with cardiac remodeling and endothelial dysfunction [35,36]. At present, their use in HF is limited by assay variability and insufficient clinical validation. Overall, liver involvement in HF reflects the interaction between hemodynamic stress and molecular pathways responsible for inflammation, iron regulation, metabolic signaling and fibrosis. This explains why liver-derived biomarkers and hepatic scores may carry prognostic information beyond isolated liver injury. In advanced HF, they may reflect not only congestion or hypoperfusion, but also systemic severity and reduced physiological reserve.

## 5. Classical Liver-Derived Biomarkers in Heart Failure

In bedside practice, liver-derived biomarkers can be grouped into three distinct clinical profiles that reflect the dominant pathophysiological driver of each: the congestive/cholestatic, the hypoperfusive/ischemic and the mixed/systemic reserve profile.

Routine biochemical assessment remains important in HF because it provides readily available information on congestion, perfusion, renal function and systemic reserve [1,2]. However, classical liver tests have limited specificity. In patients with HF, abnormalities in bilirubin, cholestatic enzymes, aminotransferases, albumin and coagulation parameters should not be interpreted in isolation as evidence of liver disease [4,13]. Their clinical value depends on the combination of abnormalities, their severity, their evolution during treatment, and their relationship with renal function, sodium concentration, congestion status, and anticoagulant therapy [5,8]. Bilirubin is one of the most informative liver-derived parameters in HF. Its concentration may increase as a result of impaired sinusoidal drainage, reduced hepatocyte clearance and disturbed bile transport [4,13]. When hyperbilirubinemia is accompanied by increased GGT or ALP, it usually supports a cholestatic component of congestive hepatopathy rather than primary hepatocellular injury [5,12]. However, bilirubin is not specific to HF. Gilbert syndrome, hemolysis, biliary obstruction, alcohol-related liver injury, metabolic liver disease, drug-induced liver injury and primary cholestatic disorders should be considered when abnormalities are disproportionate to the clinical picture or persist despite hemodynamic improvement [4,13]. GGT and ALP provide additional information on cholestatic involvement. When GGT or ALP is elevated in isolation, alcohol use, metabolic liver disease, cholestatic disorders and medications should be considered [13]. Aminotransferases provide different clinical information. Mild aspartate aminotransferase (AST) or alanine aminotransferase (ALT) elevation may occur in chronic congestion, but a rapid and marked increase should raise suspicion of acute hepatocellular injury related to low CO, shock or severe circulatory deterioration [4,14]. In hypoxic hepatitis, aminotransferase elevation is usually abrupt and may be accompanied by high lactate dehydrogenase (LDH) activity, worsening renal function, metabolic acidosis, or elevated lactate [14]. This biochemical profile should prompt urgent assessment of systemic perfusion, oxygenation and hemodynamic stability [14]. AST interpretation requires particular caution because it may also originate from skeletal muscle or the myocardium, especially in critically ill patients [13].

Albumin has a broader clinical meaning than most liver-derived parameters. Although it may reflect hepatic synthetic function, in HF it more often represents the combined effects of inflammatory activation, impaired nutritional status, fluid overload with hemodilution, renal loss, and GI protein loss [11]. Hypoalbuminemia is clinically relevant because it is associated with edema, frailty, cachexia, and adverse outcomes [6,11]. Coagulation parameters may reflect hepatic synthetic function, but their interpretation is difficult in CV populations [4,13]. INR may increase in severe hepatic injury, vitamin K deficiency or advanced systemic illness [13]. However, many patients with HF receive oral anticoagulation because of atrial fibrillation (AF), mechanical valves, or LVAD therapy [9,15]. In these patients, INR is treatment-dependent and cannot be regarded as a reliable marker of liver function [9,16]. This limitation is one of the main reasons for the use of MELD-XI, which excludes INR and may be more applicable than standard MELD in anticoagulated HF populations [9,16]. Creatinine and sodium are essential for the interpretation of liver-related abnormalities in HF. Creatinine reflects renal dysfunction, which may result from impaired forward flow, venous congestion, neurohormonal activation, CKD, or diuretic therapy [1,41]. Sodium reflects neurohormonal activation, water retention and advanced HF physiology [42]. Their inclusion in MELD, MELD-XI, MELD-Na and MELD-Albumin is clinically justified since hepatic abnormalities in HF rarely occur in isolation [16,43]. Classical biomarkers should therefore be interpreted serially rather than on the basis of a single measurement, because changes over time may suggest whether end-organ congestion is reversible [5,8]. This is particularly important in patients considered for LVAD therapy or heart transplantation, where the reversibility of hepatic and renal dysfunction may influence treatment strategy and perioperative risk [9,15].

The clinical interpretations of the main liver-related laboratory parameters are summarized in Table 2.

## 6. Composite Liver-Related Scores in Heart Failure

The utility of composite liver-related scores in HF stems from their integration of routinely available laboratory parameters into a broader estimate of systemic risk [9,16]. In advanced HF, these scores may reflect multiorgan dysfunction [1,44]. The use of such scores is consistent with the general approach proposed in ISHLT recommendations for heart transplant candidate evaluation, which emphasize assessment of extracardiac organ dysfunction and its potential reversibility [44]. However, MELD, MELD-XI, MELD-Na, MELD-Albumin and ALBI should not be treated as independent eligibility criteria for LVAD implantation or heart transplantation. Their role is supportive and should be integrated with clinical assessment, echocardiography, invasive hemodynamics and evaluation of renal and hepatic reserve [9,15,44]. The MELD score was developed to estimate prognosis in advanced liver disease and was later adopted for liver transplant allocation [45,46]. It includes bilirubin, creatinine and INR [45,46]. The main limitation of standard MELD in CV populations is the inclusion of INR, which is frequently affected by oral anticoagulation [9,47]. Modified MELD scores have been proposed in CV populations mainly to reduce the confounding effect of anticoagulation [48,49,50]. MELD-XI was developed to avoid this limitation by excluding INR and using only bilirubin and creatinine [47]. In patients with advanced HF evaluated for heart transplantation, MELD-XI provides prognostic information and may help identify patients at increased perioperative or post-transplant risk [17,48,49]. MELD-Na adds serum sodium to the standard MELD score [43]. This modification is relevant in HF as hyponatremia reflects neurohormonal activation, impaired free-water clearance, and advanced disease physiology [42]. At the same time, MELD-Na retains the limitations related to INR and is influenced by diuretic therapy, fluid restriction and acute changes in volume status [1,41,42].

In MELD-Albumin, the INR used in the standard MELD score is replaced by albumin [50]. This approach is clinically reasonable given that albumin reflects hepatic synthesis, inflammatory activation, nutritional status, and systemic reserve [11,50]. However, albumin is also influenced by fluid overload with hemodilution, renal loss, GI protein loss and malnutrition [11]. The ALBI score, originally developed to assess liver function in patients with hepatocellular carcinoma (HCC), combines bilirubin and albumin [18]. Although ALBI was not developed for HF, it has been evaluated in independent cohorts of hospitalized patients with HF [10] and patients with acute HF [51,52], as well as in decompensated-HF and LVAD populations [53,54]. However, the reported cut-offs are cohort-specific and require further validation before they can be applied more broadly in HF.

Other liver-related scores have also been explored in HF. The Fibrosis-4 (FIB-4) and Fibrosis-5 (FIB-5) indices, originally developed as non-invasive markers of liver fibrosis, have shown prognostic value in selected HF populations [55,56]. In HF, however, their prognostic significance may reflect not only hepatic fibrosis but also congestion and systemic disease, and their clinical utility requires further validation. A more recent development is MELD 3.0, which was designed to improve mortality prediction in liver transplant candidates and incorporates bilirubin, creatinine, INR, sodium, albumin and female sex [57]. Evidence in HF is currently limited mainly to patients with advanced HF undergoing heart transplantation. In a cohort of 106 heart transplant recipients, postoperative, but not preoperative, MELD 3.0 was independently associated with short-term mortality, indicating potential prognostic value that requires further validation [58].

The main composite liver-related scores are presented in Table 3.

## 7. Advanced Heart Failure, LVAD Therapy and Heart Transplantation

In advanced HF, liver-related abnormalities are clinically significant primarily due to their utility in evaluating organ reserve and the potential reversibility of hepatorenal dysfunction prior to advanced therapies [1,2]. Abnormalities in bilirubin, cholestatic enzymes, albumin, creatinine or liver-related scores should therefore be interpreted in relation to RV function, pulmonary pressures, systemic congestion, renal function, nutritional status, and current treatment [5,8,11]. In patients considered for LVAD therapy or heart transplantation, the key clinical question is whether these abnormalities reflect reversible hemodynamic dysfunction or more advanced organ injury [9,15].

This approach is consistent with ISHLT recommendations, which emphasize assessment of extracardiac organ dysfunction and its potential reversibility [44]. Laboratory abnormalities related to congestion or low CO may improve after decongestion, inotropic support, temporary mechanical circulatory support (tMCS) or ventricular unloading [5,9,15]. Conversely, sustained hyperbilirubinemia, progressive hypoalbuminemia, worsening renal function, or persistently elevated liver-related scores despite optimized treatment may suggest reduced hepatorenal reserve [16,48]. For this reason, liver-derived biomarkers and liver-related scores may support risk stratification, but they should not be used as independent eligibility criteria for LVAD implantation or heart transplantation [44].

Before LVAD implantation, cardiohepatic assessment is particularly relevant, since postoperative outcome depends substantially on RV function [15,60]. LVAD therapy improves left-sided unloading and systemic forward flow, but persistent or worsening RV dysfunction may maintain systemic venous congestion after implantation [15,60]. In this setting, preoperative abnormalities in bilirubin, creatinine, MELD or MELD-XI may identify patients with limited hepatorenal reserve and higher perioperative risk [9,15,60]. The interpretation of INR-based scores warrants caution, given the high prevalence of anticoagulation among LVAD candidates and recipients [9,47]. INR-independent approaches, including MELD-XI, MELD-Albumin and ALBI, may be easier to interpret, but they still require clinical correlation and HF-specific validation [9,18,47,53,54].

In clinical practice, differentiating reversible congestive hepatopathy from advanced structural liver disease remains challenging [12,66]. Routine liver tests may underestimate fibrosis, whereas imaging and elastography may be confounded by congestion [66,67]. Persistent liver abnormalities, suspected cirrhosis, portal hypertension, ascites disproportionate to cardiac congestion, or unclear hepatic disease should prompt a hepatology consultation [44,66]. In selected patients, further evaluation may include liver imaging, elastography, assessment for portal hypertension, or liver biopsy [48,66].

Thus, in advanced HF, liver-derived biomarkers and hepatic scores are most useful when interpreted serially and in close relation to RV function, hemodynamic status and treatment response [5,9,44]. Their value lies in supporting individualized risk assessment and multidisciplinary decision-making, rather than replacing comprehensive clinical evaluation [44].

The available evidence comes from clinically heterogeneous HF populations and should not be considered directly interchangeable. Studies in acute or hospitalized HF assessed in-hospital and longer-term mortality and, in some cases, serial changes during treatment [10,50,51,52,61], whereas ambulatory advanced HF cohorts focused on longer-term outcomes and the need for advanced therapies [16,64]. Studies involving severe TR, LVAD candidates and recipients, and heart transplant recipients reflect different hemodynamic mechanisms, treatments and competing risks [9,15,49,60,62,65]. Dedicated evidence in predominant RV failure and pulmonary hypertension remains limited. Data stratified by HF phenotype are also scarce, with HF with preserved ejection fraction (HFpEF) evaluated separately in only a limited number of studies [63]. In addition, concomitant MASLD or other chronic liver disease may also complicate biomarker and score interpretation and should be considered when distinguishing primary liver disease from cardiohepatic injury [12,39,40,66].

Some studies showed that liver-related scores provided additional prognostic information beyond conventional clinical variables, the N-terminal prohormone of brain natriuretic peptide (NT-proBNP), or hemodynamic measures [10,51,63,64]. Other studies reported performance comparable to established LVAD or RV-failure models [15,60]. However, because creatinine is included in several of these scores, their added value beyond renal function alone remains uncertain [9,16,47]. Most analyses were internal, and external validation was generally lacking [10,15,16,51,52,53,54,60,63,64]. None of the studies summarized in Table 4 assessed whether score-guided management improves clinical outcomes. These findings support the prognostic relevance of these scores but do not establish their clinical utility.

## 8. Clinical Integration and Future Directions

In advanced HF, abnormal liver-related test results should be interpreted in the context of the dominant hemodynamic profile. Increased bilirubin and cholestatic enzymes may reflect venous congestion, whereas a marked rise in aminotransferases may indicate hypoperfusion or acute circulatory deterioration [4,12,14]. Albumin, creatinine and sodium provide additional information on inflammatory and nutritional status, cardiorenal dysfunction, neurohormonal activation and systemic reserve [11,41,42]. Their interpretations should also consider RV function, TR, pulmonary pressures, volume status and current treatment [5,8,44].

Future studies should assess the clinical value of serial measurements of liver-related biomarkers and scores in well-defined populations, including patients with acute decompensated HF, advanced ambulatory HF, LVAD candidates and recipients, and heart transplant candidates [8,44]. Furthermore, future investigations should clarify how laboratory scores, liver stiffness, imaging, assessment of portal hypertension, and invasive hemodynamics can be combined to identify clinically relevant and potentially irreversible hepatic injury [44,66,67].

Artificial intelligence (AI) may help integrate these different types of data. In the study by Gao et al., a model using admission notes and structured clinical data predicted in-hospital outcomes more accurately than models based on either source alone [69]. However, its clinical usefulness still needs to be confirmed in independent prospective studies.

Beyond standard biochemistry, several novel liver-related molecular markers may improve the understanding of cardiohepatic injury [19,25]. Hepcidin links iron regulation with inflammation-driven functional iron deficiency, although its specific contribution in HF remains uncertain [21,25,26]. Bile acid-related mediators and hepatokines are connected with metabolic, inflammatory and cardiometabolic pathways [29,30,31,32,33]. Extracellular vesicles and microRNAs may participate in intercellular and interorgan communication, but their clinical use in HF remains insufficiently validated [34,35,36]. At present, these markers should be regarded as investigational rather than clinically established. Furthermore, clinical risk models should increasingly incorporate the principles of CKLMS and MALCKS to capture systemic metabolic reserve. Evaluating liver biomarkers within these multi-organ frameworks could enable earlier identification of patients with combined metabolic, renal, and hepatic vulnerability, further refining therapeutic strategies [39,40].

**Figure 2:**
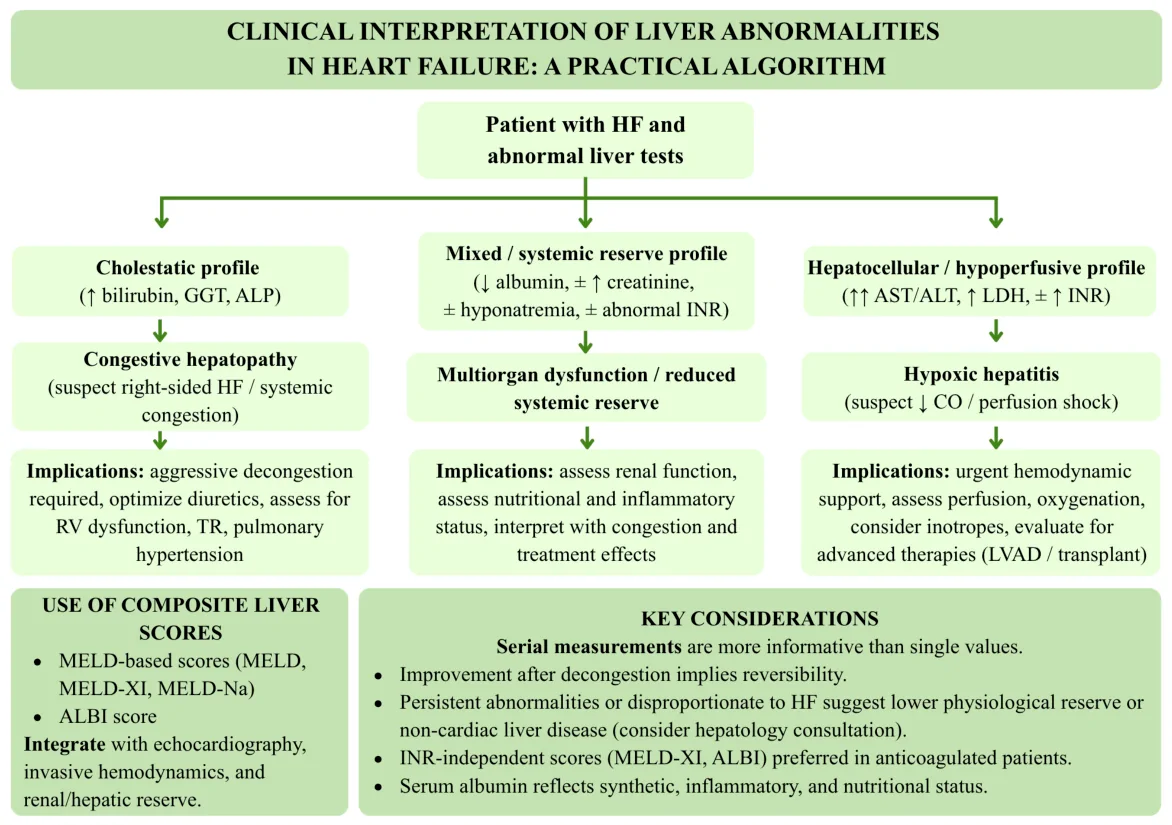
Clinical interpretation of liver abnormalities in heart failure: a practical algorithm. Downward green arrows indicate the step-by-step clinical decision-making flow from biochemical profiling to diagnostic suspicion and clinical management. Upward (↑) and downward (↓) arrows within the boxes represent elevated and decreased parameters, respectively. Abbreviations: ALBI—Albumin–bilirubin score; ALP—Alkaline phosphatase; ALT—Alanine aminotransferase; AST—Aspartate aminotransferase; CO—Cardiac output; GGT—Gamma-glutamyl transferase; HF—Heart failure; INR—International Normalized Ratio; LDH—Lactate dehydrogenase; LVAD—Left ventricular assist device; MELD—Model for End-Stage Liver Disease; MELD-Na—MELD with sodium; MELD-XI—MELD excluding INR; RV—Right ventricular; TR—Tricuspid regurgitation.

Taken together, these observations support the concept of the liver as a biomarker organ in HF. Figure 2 summarizes the proposed approach to the interpretation of liver-related abnormalities.

## 9. Conclusions

Liver-related laboratory abnormalities remain an underrecognized component of HF assessment. They rarely indicate isolated hepatic injury, but instead reflect the combined effects of venous congestion, impaired forward flow, renal dysfunction, inflammation, nutritional status and reduced physiological reserve. Bilirubin and cholestatic enzymes may support the recognition of systemic congestion, particularly in patients with RV dysfunction or severe TR, whereas marked aminotransferase elevations should raise suspicion of hypoperfusion or acute circulatory deterioration. Albumin, INR, creatinine and sodium provide additional information on multiorgan involvement, but require interpretation in relation to treatment and the patient’s clinical and hemodynamic profile.

Composite liver-related scores can help organize these abnormalities and identify patients with greater cardiohepatic and hepatorenal vulnerability. MELD-XI and ALBI may be particularly useful when INR is affected by anticoagulation. Their greatest potential in HF lies in serial assessment and integration with RV function, congestion, renal function and response to decongestion or mechanical unloading. Improvement may support a reversible cardiohepatic component, whereas persistent abnormalities may indicate reduced organ reserve and the need for more detailed evaluation, particularly before LVAD implantation or heart transplantation. These scores should support, rather than replace, comprehensive clinical assessment. Although their prognostic relevance has been demonstrated in several HF populations, further prospective studies are needed to define HF-specific thresholds, confirm their added value and determine whether their use can improve clinical decision-making. The concept of the liver as a biomarker organ may therefore broaden clinical assessment beyond cardiac function alone.

## Figures and Tables

**Table 1:** Hemodynamic and systemic mechanisms of cardiohepatic injury in heart failure and their laboratory correlates.

Mechanism	Main Abnormality	Typical Laboratory Profile	Clinical Interpretation	Important Limitations
Venous congestion [2,4,5,12]	Increased right atrial pressure transmitted to the inferior vena cava, hepatic veins and sinusoids.	Increased bilirubin, GGT, and ALP; aminotransferases usually normal or mildly elevated.	Suggests right-sided HF, severe TR, pulmonary hypertension or systemic venous congestion.	Liver tests may remain normal despite clinically relevant congestion; cholestatic abnormalities may also reflect primary liver or biliary disease.
Reduced forward flow [2,4,14]	Reduced cardiac output with impaired hepatic oxygen delivery.	Increased aminotransferases; in severe cases high LDH, increased INR and bilirubin.	Suggests low-output state, shock, severe acute decompensation or hypoxic hepatitis.	May occur without profound systemic hypotension, especially when venous congestion is present.
Combined congestion and hypoperfusion [2,5,8,14]	Elevated venous pressure with reduced effective hepatic perfusion.	Mixed abnormalities involving bilirubin, aminotransferases, cholestatic enzymes, INR, albumin and creatinine.	Common in advanced HF and usually indicates more severe systemic and multiorgan involvement.	The dominant mechanism cannot usually be defined from laboratory tests alone.
RV dysfunction [2,5,9,12,15]	Increased systemic venous pressure due to impaired RV function.	Predominantly congestive profile; bilirubin and cholestatic enzymes may predominate.	Important in advanced HF, severe TR, pulmonary hypertension and pre-LVAD or transplant assessment.	Requires echocardiographic and, in selected patients, invasive hemodynamic assessment.
Inflammatory activation and impaired nutritional status [1,6,8,11]	Inflammatory activation, catabolism, fluid overload with hemodilution and impaired nutritional status.	Low albumin, anemia, iron deficiency and increased inflammatory markers.	Indicates frailty, inflammatory activation, impaired nutritional status and reduced systemic reserve.	Hypoalbuminemia should not be interpreted automatically as hepatic failure.
Hepatorenal dysfunction [9,10,16,17,18]	Venous congestion and/or hypoperfusion affecting both liver and kidneys.	Increased creatinine with hyperbilirubinemia, hyponatremia or abnormal liver-related scores.	Supports integrated risk assessment with MELD, MELD-XI and MELD-Na; ALBI reflects the albumin–bilirubin component.	Composite scores summarize risk but do not identify one specific mechanism of liver injury.

Abbreviations: ALP—Alkaline phosphatase; ALBI—Albumin-bilirubin score; GGT—Gamma-glutamyl transferase; HF—Heart failure; INR—International Normalized Ratio; LDH—Lactate dehydrogenase; LVAD—Left ventricular assist device; MELD—Model for End-Stage Liver Disease; MELD-XI—Model for End-Stage Liver Disease Excluding INR; MELD-Na—Model for End-Stage Liver Disease with Sodium; RV—Right ventricular; TR—Tricuspid regurgitation.

**Table 2:** Interpretation of liver-related laboratory parameters in heart failure.

Biomarker	Main Interpretation in HF	Clinical Relevance	Important Limitations	Clinical Profile
Bilirubin [5,6,8,12,13]	Reflects venous congestion, impaired sinusoidal drainage, reduced hepatic clearance and disturbed bile transport.	May indicate congestion-related liver involvement and higher systemic risk.	Not specific; may increase in Gilbert syndrome, hemolysis, biliary obstruction, drug-induced liver injury, or primary liver disease.	Congestive/Cholestatic
GGT [5,8,13]	May reflect congestion-related cholestasis and impaired bile transport.	Supports cholestatic liver involvement when accompanied by hyperbilirubinemia and clinical congestion.	Influenced by alcohol use, metabolic liver disease, cholestatic disorders and medications.	Congestive/Cholestatic
ALP [5,12,13]	Indicates cholestasis or impaired bile flow; may increase in chronic hepatic congestion.	May support congestion-related cholestatic involvement, especially when assessed together with bilirubin and GGT.	Requires exclusion of biliary obstruction, cholestatic liver disease and bone-related causes.	Congestive/Cholestatic
AST/ALT [4,12,14]	Reflect hepatocellular injury; marked elevation suggests hypoxic hepatitis or acute circulatory deterioration.	Useful for identifying severe low-output states, shock or acute worsening of systemic perfusion.	Mild elevations are nonspecific; AST may also originate from skeletal muscle or myocardium.	Hypoperfusive/Ischemic
Albumin [6,8,11]	Reflects hepatic synthetic function, but also inflammatory activation, impaired nutritional status, fluid overload with hemodilution, and protein loss.	Low albumin may indicate frailty, edema and reduced systemic reserve.	Should not be interpreted as liver failure without clinical context; influenced by renal and GI protein loss.	Mixed/Systemic Reserve
INR [9,13,16]	May reflect impaired hepatic synthesis of coagulation factors in non-anticoagulated patients.	Included in standard MELD and may contribute to assessment of hepatic synthetic dysfunction.	Limited in HF because oral anticoagulation is common; affected by vitamin K status, drugs and systemic illness.	Mixed/Systemic Reserve
Creatinine [16,41]	Reflects renal dysfunction as part of the hepatorenal and hemodynamic profile.	Central component of MELD and MELD-XI; helps identify combined hepatic and renal risk.	Influenced by age, sex, muscle mass, CKD, diuretic therapy and acute hemodynamic changes.	Mixed/Systemic Reserve
Sodium [42,43]	Reflects neurohormonal activation, impaired water handling and advanced HF physiology.	Relevant in advanced HF and incorporated into MELD-Na as a marker of systemic severity.	Influenced by diuretics, fluid intake, congestion status and treatment strategy.	Mixed/Systemic Reserve

Abbreviations: ALP—Alkaline phosphatase; ALT—Alanine aminotransferase; AST—Aspartate aminotransferase; CKD—Chronic kidney disease; GGT—Gamma-glutamyl transferase; GI—Gastrointestinal; HF—Heart failure; INR—International Normalized Ratio; MELD—Model for End-Stage Liver Disease; MELD-Na—Model for End-Stage Liver Disease with Sodium; MELD-XI—Model for End-Stage Liver Disease Excluding INR.

**Table 3:** Composite liver-related scores used or proposed in heart failure.

Score	Formula and Laboratory Units	Dialysis and Extreme Values	Original Study Population	Evidence in HF: Populations, Outcomes and Reported Cut-Offs	Key Confounders and Limitations
MELD [45,46]	3.78 × ln[bilirubin (mg/dL)] + 11.2 × ln(INR) + 9.57 × ln[creatinine (mg/dL)] + 6.43.	Values < 1.0 are set to 1.0. Creatinine is capped at 4.0 mg/dL and set to 4.0 in patients undergoing dialysis at least twice during the preceding week. In liver allocation, the final score is bounded from 6 to 40.	Developed in patients with cirrhosis undergoing TIPS [59], subsequently validated in broader cohorts with end-stage liver disease [45], and later adopted for liver allocation [46].	Evaluated in ambulatory advanced HF, LVAD and transplant cohorts. In ambulatory advanced HF, MELD > 12 identified a higher-risk group; in earlier VAD cohorts, a cut-off of 17 was used [9,16].	INR is affected by anticoagulation, vitamin K status and systemic illness. Creatinine is influenced by sarcopenia, CKD, diuretic treatment and acute hemodynamic changes. The score is not specific to cardiohepatic injury.
MELD-XI [47]	5.11 × ln[bilirubin (mg/dL)] + 11.76 × ln[creatinine (mg/dL)] + 9.44.	Bilirubin and creatinine values below 1.0 mg/dL are set to 1.0; creatinine values above 4.0 mg/dL are capped at 4.0, and creatinine is set to 4.0 mg/dL in patients receiving dialysis [47]. Some HF studies used different handling rules, which should therefore be reported.	Derived in patients with cirrhosis requiring oral anticoagulation and validated in liver-transplant candidate cohorts.	Evaluated in acute and ambulatory HF, HFpEF, severe TR, LVAD and transplant populations [9,15,16,17,49,60,61,62,63]. Reported cut-offs include >9.44 in HFpEF [63], ≥12 in patients undergoing tricuspid annuloplasty [62], ≥14 in LVAD cohorts [15,60], and ≥17 in an earlier VAD cohort [9].	Strongly influenced by renal dysfunction and bilirubin elevation from non-cardiac causes. It does not account for albumin, sodium, frailty, inflammation or nutritional status.
MELD-Na (original Biggins et al. formula) [43]	MELD + 1.59 × [135 − sodium (mmol/L)]. This is the historical formula used in the cited HF literature; later liver-allocation formulas are not identical.	Sodium is bounded between 120 and 135 mmol/L. The laboratory limits and dialysis rule used for the underlying MELD score also apply.	Derived in adult liver-transplant waiting-list candidates to improve prediction of waiting-list mortality.	Evaluated mainly in ambulatory advanced HF and acute HF. MELD-Na > 12 was associated with an increased risk of adverse outcomes at 1 year in an ambulatory transplant-evaluation cohort [16].	Retains the limitations related to INR. Sodium is affected by diuretics, fluid intake, decongestion and acute changes in water balance. Results depend on the specific MELD-Na formula used.
Albumin-substituted MELD variants: modMELD/MELD-A/MELD-Albumin [50,62,64,65]	If albumin ≥ 4.1 g/dL: 11.2 × ln(1) + 3.78 × ln[bilirubin (mg/dL)] + 9.57 × ln[creatinine (mg/dL)] + 6.43. If albumin < 4.1 g/dL: 11.2 × ln [1 + (4.1 − albumin (g/dL))] + 3.78 × ln[bilirubin (mg/dL)] + 9.57 × ln[creatinine (mg/dL)] + 6.43.	Bilirubin and creatinine values < 1.0 mg/dL are set to 1.0. Creatinine values > 4.0 mg/dL are commonly capped at 4.0. The handling of dialysis has varied, and patients undergoing dialysis were excluded from the principal AHF study [50].	Introduced in heart-transplant recipients as modified MELD (modMELD), with albumin replacing INR [65], and subsequently evaluated as MELD-A/MELD-Albumin in HF populations [50,62,64].	Evaluated in ambulatory advanced HF, AHF and patients undergoing tricuspid valve surgery [50,62,64]. Reported cut-offs include >14 in ambulatory advanced HF [64] and ≥10.7 in patients undergoing tricuspid annuloplasty [62].	Albumin is affected by inflammation, malnutrition, renal or gastrointestinal protein loss, and hemodilution.
ALBI [10,18,51,52,53,54]	0.66 × log_10_[bilirubin (μmol/L)] − 0.085 × albumin (g/L). Original grades: grade 1, ≤−2.60; grade 2, >−2.60 to ≤−1.39; grade 3, >−1.39.	The original model does not specify a dialysis rule or truncation of extreme values. The continuous score and the timing of laboratory measurements should be reported.	Derived in patients with hepatocellular carcinoma across different disease stages [18].	Evaluated in acute and decompensated HF and in LVAD cohorts. Reported cut-offs include −2.32 for in-hospital mortality [10], −2.25 for in-hospital mortality [52], and −2.191 for long-term mortality [53]. Matsue et al. did not propose a cut-off [51].	Albumin and bilirubin are affected by inflammation, nutritional status, fluid balance, hemolysis, cholestasis and primary liver disease. Hepatology-derived grades have not been validated as treatment thresholds in HF.

Abbreviations: AHF—Acute heart failure; ALBI—Albumin–bilirubin score; CKD—Chronic kidney disease; HF—Heart failure; HFpEF—Heart failure with preserved ejection fraction; INR—International Normalized Ratio; LVAD—Left ventricular assist device; MELD—Model for End-Stage Liver Disease; MELD-Na—Model for End-Stage Liver Disease with Sodium; MELD-XI—Model for End-Stage Liver Disease Excluding INR; TIPS—Transjugular intrahepatic portosystemic shunt; TR—Tricuspid regurgitation; VAD—Ventricular assist device.

**Table 4:** Studies evaluating the prognostic value of composite liver-related scores in heart failure and advanced cardiovascular populations.

Study and Population	Score and Assessment Time	Outcome and Follow-Up	Main Findings and Reported Cut-Off	Validation and Main Limitations
Chokshi et al., 2012 [65]; *n* = 617 adult heart-transplant recipients.	MELD and modMELD before transplantation.	Thirty-day complications and in-hospital death; long-term survival.	In-hospital mortality was 16% with MELD >20 versus 3% with MELD <14, and 21% with modMELD >20 versus 5% with a score <14. Higher scores were associated with poorer long-term survival.	Single-center retrospective cohort; no external validation; limited hemodynamic and hepatic data.
Yang et al., 2012 [9]; *n* = 255 patients receiving pulsatile- or continuous-flow VAD support.	MELD and MELD-XI before implantation and during VAD support.	On-VAD, post-transplant and overall survival.	Scores < 17 were associated with better on-VAD and overall survival. Normalization of an initially elevated MELD-XI during support was associated with post-transplant survival similar to that of the low-score group.	Single-center retrospective cohort; no external validation; older devices and a cohort-derived cut-off.
Kim et al., 2013 [16]; 343 patients were evaluated; the complete-case analytical cohort included 260 patients with advanced ambulatory HF.	MELD, MELD-Na and MELD-XI at evaluation.	Death, heart transplantation or VAD requirement within 1 year.	AUC 0.71 for MELD and 0.73 for MELD-Na; HR 1.10 (95% CI 1.06–1.14) per point for both scores; reported cut-off >12.	Single-center retrospective cohort; no external validation; INR-based scores may be affected by anticoagulation.
Kato et al., 2013 [64]; *n* = 151 ambulatory patients with advanced HF undergoing transplant evaluation.	MELD-A at evaluation, combined with exercise and hemodynamic variables.	Death, urgent heart transplantation or VAD implantation within 1 year.	MELD-A > 14 was independently associated with events: OR 3.72 (95% CI 1.48–9.36). A combined model including peak VO_2_, PCWP and RVSWI had an AUC of 0.790.	Small single-center retrospective cohort; internally derived model and cut-off; no external validation.
Biegus et al., 2016 [61]; *n* = 203 patients hospitalized with acute HF.	MELD-XI on admission and on days 2–3.	All-cause mortality at 1 year; 67 deaths (33%).	Adjusted HR per point: 1.11 (95% CI 1.05–1.20) at baseline and 1.14 (1.09–1.20) during admission. A rise in MELD-XI, observed in 31%, was associated with HR 1.97 (1.2–3.2).	Small single-center cohort; no validated cut-off or external validation.
Deo et al., 2016 [49]; *n* = 36,005 adult heart-transplant recipients with calculable MELD-XI in the UNOS registry.	Pre-transplant MELD-XI, analyzed continuously and by quartiles.	Mortality and post-transplant morbidity; median follow-up 8.1 years.	Adjusted HR 1.021 (95% CI 1.016–1.026) per point for mortality. Highest versus lowest quartile: HR 1.364 (1.255–1.482). Higher scores were also associated with major postoperative complications.	Large retrospective registry; quartile-based comparisons; no assessment of clinical utility.
Chen et al., 2018 [62]; *n* = 394 patients undergoing elective tricuspid annuloplasty.	Preoperative MELD-XI and MELD-Albumin.	HF admission or all-cause death; median follow-up 40 months.	One-year AUC was 0.69 for MELD-XI (cut-off 12.0) and 0.75 for MELD-Albumin (cut-off 10.7). For 1-year adverse events, the adjusted HR per point was 1.13 (95% CI 1.06–1.21) for MELD-XI and 1.13 (95% CI 1.07–1.20) for MELD-Albumin.	Single-center retrospective surgical TR cohort; no external validation.
Critsinelis et al., 2018 [15]; *n* = 524 HeartMate II or HeartWare LVAD recipients.	Preoperative MELD-XI; <14 versus ≥14.	Mortality up to 24 months; early RV failure and infection.	MELD-XI ≥ 14 was associated with lower survival at all reported time points (all *p* < 0.001), as well as more early RV failure and infections. Performance for 90-day mortality was comparable with the HeartMate II Risk Score.	Single-center retrospective cohort; older-generation devices; no external validation.
Matsue et al., 2020 [51]; *n* = 1190 patients with acute HF.	ALBI and MELD-XI at admission.	All-cause mortality at 1 year.	The adjusted HR for 1-year mortality was 2.11 (95% CI 1.60–2.79) per 1-unit higher ALBI. Adding ALBI to established prognostic factors increased the AUC from 0.71 to 0.74 (*p* = 0.020); no cut-off was proposed.	Observational registry analysis; no external validation or validated cut-off.
Kawata et al., 2021 [52]; *n* = 262 patients hospitalized with acute HF.	ALBI at admission.	In-hospital all-cause mortality.	In-hospital mortality was 21.1% with ALBI >−2.25 versus 4.5% with ALBI ≤−2.25 (*p* = 0.0001); adjusted OR 6.0 (95% CI 1.8–19.6).	Small, single-center cohort of predominantly elderly patients; internally derived cut-off and no external validation.
Liao et al., 2021 [50]; *n* = 466 patients with acute HF.	MELD, MELD-XI, MELD-Na and MELD-Albumin at admission.	All-cause mortality; median follow-up 34 months.	Adjusted HR per 1 SD was 1.22 (95% CI 1.06–1.40) for MELD, 1.20 (1.04–1.39) for MELD-XI, 1.23 (1.06–1.42) for MELD-Na and 1.21 (1.05–1.41) for MELD-Albumin. The corresponding four-year AUCs were 0.580, 0.544, 0.590 and 0.658. MELD-Albumin improved NRI by 11.9–14.8% compared with the other scores.	Prospective multicenter cohort; modest discrimination; no external validation.
Wang et al., 2021 [63]; *n* = 30,096 hospitalized patients with HFpEF.	MELD-XI at admission; elevated score defined as >9.44.	Sixty-day in-hospital all-cause and cardiovascular mortality and 30-day HF readmission.	Adjusted HR per point: 1.052 (95% CI 1.022–1.083) for all-cause death, 1.064 (1.013–1.118) for cardiovascular death and 1.061 (1.015–1.108) for readmission. Model AUC increased from 0.858 to 0.868.	Single-center retrospective cohort; short follow-up; no external validation. The >9.44 value was not a validated treatment threshold.
Han et al., 2022 [10]; *n* = 9749 patients hospitalized with HF.	ALBI at admission.	In-hospital mortality.	Adjusted OR 1.082 (95% CI 1.052–1.114) per 0.1-point increase; AUC 0.650 (0.641–0.660); cut-off −2.32, sensitivity 0.632 and specificity 0.630. Adding ALBI to NT-proBNP improved reclassification.	Retrospective internal analysis; no external validation; ALBI may be affected by inflammation, nutrition and hemodilution.
Lambert et al., 2025 [60]; *n* = 246 primary HeartMate 3 recipients.	MELD-XI before implantation; threshold ≥14.	Severe post-LVAD RV failure, which occurred in 74 patients (30%), and in-hospital mortality.	Adjusted OR 1.18 (95% CI 1.09–1.29) per point for severe RV failure. MELD-XI ≥14 was also associated with higher in-hospital mortality and performed similarly to established RV-failure scores.	Two-center retrospective cohort; no independent validation.
Jurkiewicz et al., 2025 [53]; *n* = 242 patients aged >65 years hospitalized with worsening chronic HF.	ALBI at admission.	All-cause mortality; mean follow-up 352 ± 293 days.	AUC 0.822 (95% CI 0.768–0.875); cut-off ≥ −2.191, sensitivity 68% and specificity 86%; high versus low ALBI: HR 4.42 (2.97–6.58).	Small single-center retrospective cohort; internally derived threshold; no external validation.
Niklewski et al., 2025 [54]; *n* = 95 HeartMate 3 recipients.	ALBI before implantation.	All-cause mortality; median follow-up 835 days.	Adjusted weighted Cox HR 4.981 (95% CI 2.791–8.890) per 1-unit higher ALBI (*p*< 0.001).	Very small single-center retrospective cohort; no validated cut-off or external validation.
Salman et al., 2026 [68]; meta-analysis of nine observational studies including 33,316 patients with HF.	MELD or MELD-XI; assessment time varied.	All-cause mortality; follow-up varied.	Pooled HR was 1.12 (95% CI 1.06–1.18). The MELD-XI subgroup HR was 1.09 (1.04–1.14), and the MELD subgroup HR was 1.10 (1.06–1.14); the subgroup difference was not significant (*p* = 0.70). Substantial heterogeneity was reported.	Meta-analysis of observational studies; substantial heterogeneity, inconsistencies in study classification and limited comparability across HF populations; no assessment of clinical utility.

Abbreviations: ALBI—Albumin–bilirubin score; AUC—Area under the curve; CI—Confidence interval; HF—Heart failure; HFpEF—Heart failure with preserved ejection fraction; HR—Hazard ratio; INR—International Normalized Ratio; LVAD/VAD—Left ventricular/ventricular assist device; MELD—Model for End-Stage Liver Disease; MELD-Na—MELD including sodium; MELD-XI—MELD excluding INR; NT-proBNP—N-terminal prohormone of brain natriuretic peptide; NRI—Net reclassification improvement; OR—Odds ratio; PCWP—Pulmonary capillary wedge pressure; RV—Right ventricular; RVSWI—Right ventricular stroke work index; TR—Tricuspid regurgitation; UNOS—United Network for Organ Sharing; VO_2_—Oxygen consumption.

## Data Availability

No new data were created or analyzed in this study. Data sharing is not applicable to this article.
